# History of tachinid classification (Diptera, Tachinidae)

**DOI:** 10.3897/zookeys.316.5132

**Published:** 2013-07-11

**Authors:** James E. O’Hara

**Affiliations:** 1 Canadian National Collection of Insects, Agriculture and Agri-Food Canada, 960 Carling Avenue, Ottawa, Ontario, K1A 0C6, Canada

**Keywords:** Tachinidae, Diptera, history, classification

## Abstract

The history of the classification of the Tachinidae (Diptera) is traced from Meigen to the present. The contributions of Robineau-Desvoidy, Townsend, Villeneuve, Mesnil, Herting, Wood and many others are discussed within a chronological, taxonomic, and geographic context. The gradual development of the Tachinidae into its modern concept as a family of the Oestroidea and the emergence of the classificatory scheme of tribes and subfamilies in use today are reviewed. Certain taxa that have in the past been difficult to place, or continue to be of uncertain affinity, are considered and some are given in a table to show their varied historical treatments. The more significant systematic works published on the Tachinidae in recent decades are enumerated chronologically.

## Introduction

The Tachinidae are among the largest families of Diptera with about 8500 valid species^1^. One can only guess at the true diversity of the family but at least double the number of valid species is a conservative estimate. What is not in doubt is the important ecological role these parasitoid flies play in the environment. It is desirable to organize these flies into a phylogenetically stable suprageneric classification as an aid to those who study them and to enable predictions to be made about the less studied species based on the known habits of related species.

The Tachinidae may not be the single largest family of flies on Earth but in terms of genera they tower over all of the other 140-odd families. The current number of valid genera is about 1520 (O’Hara 2012). The next largest family is Cecidomyiidae with about 760 genera and there are only two other families with more than 500 genera: Asilidae and Chironomidae (Pape et al. 2011). Taxonomically the Tachinidae are arguably the most difficult family of flies and perhaps because of this plus the size of the family and their high position on the evolutionary tree of Diptera they have received scant attention below the family level by those investigating dipteran relationships (e.g., Yeates et al. 2007, Kutty et al. 2010). There is currently an international effort aimed at addressing this imbalance by specifically targeting the Tachinidae for phylogenetic analysis using morphological and molecular data (Stireman et al. 2013).

It seems appropriate at this time to review the history of tachinid classification from its earliest beginnings, tracing how it has changed in response to discoveries of phylogenetically insightful characters and was affected by conflicting views on the nature of generic and suprageneric limits. The noticeable disharmony in the way tachinids were classified among the six biogeographic regions of the world is still in evidence today. The task that now awaits present and future tachinidologists is to determine to a better degree than in the past the evolutionary history of the Tachinidae and to classify the family in a manner than reflects its phylogeny and preserves the best elements of the most recent classifications.

## The early years

The meagre number of tachinid species known in the early 1800s was placed in about a dozen genera with the majority of them in Meigen’s (1803) broadly defined *Tachina*^2^. André-Jean-Baptiste Robineau-Desvoidy revolutionized tachinid classification with the publication of his *Essai sur les Myodaires* (Robineau-Desvoidy 1830), in which approximately 130 new genera now placed in Tachinidae were described (Evenhuis et al. 2010). Of this total, 73 genera are presently treated as valid (O’Hara 2012). Robineau-Desvoidy (1830) also proposed the name “Calypteratae” (Calyptratae) for a higher group within his Myodaria (essentially modern-day Schizophora), which with some modification in concept (most notably the addition of the Anthomyiidae) is now regarded as “one of the best established monophyletic subsections of the Schizophora” (McAlpine 1989: 1425). In this same work, Robineau-Desvoidy’s six tribes of Calypteratae approximated some of the familial and subfamilial groupings in use today in this subsection. One of these, the Entomobiae (including most of the then-known taxa of the Tachinidae), included a small number of genera grouped under the Tachinariae. The priority of the name Tachinidae over other family-group names available for this family thus dates from Robineau-Desvoidy (1830).

The *Essai sur les Myodaires* was not without its faults and received mixed reviews from dipterists of the day. Robineau-Desvoidy’s final contribution to dipterology, a massive two-volume work published in 1863 and six years after his death, *Histoire naturelle des diptères des environs de Paris*, has been justly criticized as an inferior work. In it were proposed about 160 new tachinid genera, only 25 of which are currently recognized as valid (O’Hara 2012). Similarly, a huge number of new species were described with many of them later becoming junior synonyms or *nomina dubia* (the latter resulting from the destruction of many of Robineau-Desvoidy’s name-bearing types, Evenhuis et al. 2010: 233).

Contemporaneous with Robineau-Desvoidy were Meigen, Wiedemann, Macquart and Walker, each of whom contributed significantly to the description of species but not much to the higher classification of what are now the Tachinidae. Macquart (e.g., *Diptères exotiques nouveaux ou peu connus*, 1838–1855), like Robineau-Desvoidy, recognized the need for more genera to accommodate the emerging diversity and throughout his career described about 100 tachinid genera, of which 45 are currently valid (O’Hara 2012).

The study of Diptera during the mid to late 1800s continued to be led by Europeans. Among the more notable achievements during this time were the regional treatments on the Diptera of Scandinavia by Zetterstedt (1842–1860), on Italian Diptera by Rondani (1856–1880), and on Austrian Diptera by Schiner (1860–1864). A most ambitious and influential work on the Diptera of the Vienna Museum by Brauer and Bergenstamm (1889–1895) contributed greatly to the knowledge of world Tachinidae, but was marred by an unsatisfactory and artificial suprageneric classification (e.g., Coquillett 1897, Aldrich 1905, Villeneuve 1924, Wainwright 1928, Mesnil 1944). Brauer and Bergenstamm described over 250 genera and subgenera of Tachinidae, of which 99 are currently valid genera (O’Hara 2012).

New World tachinids came under increased attention near the turn of the century, first by van der Wulp (1888–1891) and then by Coquillett (1897). Commenting on the state of tachinid classification at the time, Coquillett (1897: 27) noted:

“Probably no single family of Diptera has received greater consideration in Europe than the Tachinidae, and yet, strange as this may seem, no other family at the present time is in greater disorder. Several authors accord them only subfamily rank, but it appears desirable to consider them as a distinct family, although their relationship to the Dexidae and Sarcophagidae is a very intimate one.”

Coquillett (1897) recognized five subfamilies of Tachinidae, four representing present-day Phasiinae and one (his Tachininae) representing modern Exoristinae + Tachininae. No tribes were recognized. The “Dexidae” (Dexiidae) were regarded as a separate family and excluded.

Despite the chaotic state of tachinid classification in the late 1800s, an important methodological advance was made in the study of dipteran characters that would lead to a better understanding of natural groupings within the higher Diptera. Early authors like Meigen, Macquart and Robineau-Desvoidy had used certain large setae in their descriptions but it was Rondani (1845) who would apply the term macrochaetae (as “macrochetae”) to them. Later Osten Sacken (1881, 1884) would formalize a nomenclature for such macrochaetae under the term chaetotaxy. With refinements of the system by Girschner (1893, 1896), the study of chaetotaxy began to revolutionize the study of the more setose Diptera. Osten Sacken (1884: 511) had observed that the “hypopleural” (meral) setae “occur only in some of the Diptera Calyptrata, which have a row or a tuft of them” and Girschner (1893) used this characteristic to define the Tachinidae in the broad sense of present-day Oestroidea. Girschner also recognized several subgroups within Tachinidae *s. lat.* based on other setal arrangements. The classification was not completely satisfactory and it was only later that the enlarged subscutellum would be used to delimit the Tachinidae in a more modern sense (see below). Frey (1921) built upon the work of Girschner to further advance the classification of this group of flies.

By the beginning of the 20th Century the taxonomic literature on Palaearctic Diptera was both voluminous and daunting, especially for new students of the group. The *Katalog der paläarktischen Dipteren* (1903–1907) was therefore of immense importance, bringing together under a single classification all the names of Palaearctic Diptera. The part by Bezzi and Stein (1907) on the Schizometopa relied heavily on the work of Girschner and proposed a higher classification of considerable merit for its day. The Schizometopa were split into two families, Tachinidae and “Anthomyidae” (Anthomyiidae) (present-day Muscoidea). Within Tachinidae, ten subfamilies were recognized and listed in the following order: Tachininae, Dexiinae, Rhinophorinae, Sarcophaginae, Calliphorinae, Phasiinae, Eginiinae, “Hypoderminae” (Hypodermatinae), Oestrinae and “Gastrophilinae” (Gasterophilinae). With the exception of the Eginiinae (now placed in Muscidae), the rest of the groups with some adjustment to relative ranking closely approximates the families now recognized in the Oestroidea.

Although the *Katalog der paläarktischen Dipteren* must have been a most welcome addition to the shelf of any dipterist of the day, Mesnil (1944: 2) later criticized the Bezzi and Stein (1907) portion on the grounds that it was “voll von Irrtümern und praktisch unverwendbar” [“full of mistakes and practically unusable”].

### Classifying New World Tachinidae

North American Diptera were first catalogued by Osten Sacken (1858) and the few tachinid genera listed therein were included in the Muscidae. In the second edition of his catalogue, Osten Sacken (1878) revised the classification of Diptera and recognized both the Tachinidae and “Dexidae” (Dexiidae) as families. The next catalogue was that of Aldrich (1905), and although the Tachinidae and Dexiidae were kept separate following Osten Sacken (1878) and Coquillett (1897), the suggestion was made that they might be better combined. Aldrich (1905) followed the order of genera of Tachinidae given by Coquillett (1897) and interpolated additional genera and species as necessary. Disparaging remarks were made about the monographic works of van der Wulp (1888–1891) and Brauer and Bergenstamm (1889–1895), and of the species descriptions of Bigot (“in every way objectionable, almost always referred to the wrong genus, and seldom containing the essential data”, Aldrich 1905: 420).

Charles Henry Tyler Townsend, the most eccentric and prolific of all tachinidologists, published his first paper on tachinids in 1891 and his last in 1944, with almost 500 publications in total (the majority on tachinids) over this long period (Arnaud 1958). He took up the study of insects at the age of 10 and the study of flies at 25. He held a variety of jobs and professional appointments in the United States and later Peru before settling in Brazil for the last 25 years of his life (Townsend 1943). His most significant achievement was the *Manual of Myiology*, a 12-volume series on the “Oestromuscaria” published between 1934–1942 in which virtually every genus of these flies known at the time was placed in a suprageneric classification and given a detailed description.

Townsend was, by his own admission, a splitter of taxa. He was well versed in the works of others and offered this historical perspective on the struggle between “radicalism and conservatism” (Townsend 1935: 37):

“History shows that the taxonomy of these flies has suffered much in the making, subjected as it has been for the past century to an alternation of radicalism and conservatism, commonly called splitting and lumping. … Desvoidy, the first radical, employed restricted genera and Macquart, the first conservative, lumped them; Róndani again restricted the genera and Schiner lumped them; Brauer & Bergenstamm split, while Coquillett and Aldrich lumped; Villeneuve and Townsend again split, while Curran and Malloch lumped.”

The restricted genera of Townsend were based on the author’s concept of a “physiological genus”, defined as a “natural genus” comprising “all those species which can produce fertile crosses” (Townsend 1935: 38). As noted by van Emden (1945: 389–390), “the adoption of [this] principle implies the application of the generic unit to every unit considered to be a species in general zoological practice”. One can learn, explained Townsend (1935: 56), “to make a complete description of a fly genus and its genotype [type species] in one hour for one sex and an hour and a half for both sexes”. The ideal number of members within each of the categories of genus, tribe, family, suborder and order was set at five (Townsend 1935: 60–61). In practise Townsend rarely included more than one species per genus and throughout his career described 1491 genera and 1555 species (Arnaud 1958), with approximately 85% of the genera belonging to the Tachinidae. The number of valid tachinid genera attributed to Townsend currently stands at 544 (O’Hara 2012), more than five times that of any other author. Second place is held by Robineau-Desvoidy with 104 valid genera (O’Hara 2012).

Townsend’s methods and productivity are worth more than a cursory mention because this author has, in some ways, done more to retard tachinid taxonomy than advance it. The sheer volume of genera is one problem, and their assignment to suprageneric categories is another. Townsend knew that females of Tachinidae and related families possess a great diversity of reproductive systems that produce different kinds of eggs and larvae. After 25 years of dissecting specimens and studying the female reproductive system, he was able to recognize 36 distinct groups, most pertaining to present-day Tachinidae (Townsend 1934). Townsend (1935: 38) believed that tachinid relationships had proved to be a “Gordian knot” in the past and:

“not until the wonderful diversity of female reproductive characters and early stages was demonstrated did any sword for the cutting of this knot appear. … We are now able to determine actual relationships with greater certainty, having found the key to affinities by correlating external anatomic characters with internal reproductive and early stage characters.”

Thus armed with internal, external and larval characters, Townsend developed a unique classification that divided present-day Tachinidae among seven families (Gymnosomatidae, Oestridae, Prosenidae, Rutiliidae, Tachinidae, Dexiidae, Exoristidae) and about 90 tribes. Had this hierarchical system truly classified the Tachinidae along phylogenetic lines then it would have been the most significant advance in the history of tachinidology. However, it fell short of this goal and is now regarded as both unmanageable and artificial (e.g., Mesnil 1939, Wood 1985, 1987). Specialists also found the keys to tribes and genera in *Manual of Myiology* to be fraught with problems, thus hindering the recognition of Townsend’s supraspecific taxa.

William Robin Thompson published a series of eight papers in the *Tachinids of Trinidad* (Thompson 1961–1968). He had difficulty interpreting the fauna of Trinidad according to the Townsend scheme and chose to avoid attempting to revise Townsend’s genera:

“[I have] decided also that in most cases an attempt to simplify the taxonomic problems by reducing Townsendian genera to the synonymy is impracticable because with the knowledge we now have it is impossible to know when to stop” (Thompson 1961: 22).

Thompson found the works of Mesnil and other Europeans (see below) more helpful than the works of Townsend for understanding the major groupings of Tachinidae. Although this led Thompson to classify the Tachinidae of Trinidad in a more natural way, he had a proclivity for describing unnecessary new genera.

The tribes, genera and species created by Townsend were described predominantly for New World taxa and by their sheer number continue to pose serious challenges for taxonomists to this day. Sabrosky and Arnaud (1965), the first to catalogue the Tachinidae of America north of Mexico in the post-Townsend era, adopted a nearly modern concept of the family (differing only by the inclusion of Rhinophorinae) while otherwise retaining many of Townsend’s tribes:

“for present convenience, in the absence of any other published arrangement of the Nearctic genera, though with some combinations and generic transfers, notably where we agree with the recent work of Mesnil and coworkers in Europe. This is especially true in the Goniinae [= Exoristinae]” (p. 962).

The catalogue of the Tachinidae of America south of the United States by Guimarães (1971) followed shortly after Thompson’s *Tachinids of Trinidad* and Sabrosky and Arnaud’s catalogue. This author, faced with the huge number of tribes, genera and species described by Townsend and having to deal with other taxa inadequately described by earlier authors, could not revise the whole classification and mostly followed Townsend. This action, he admitted, resulted in a “catalogue arrangement [that] leaves much to be desired” (Guimarães 1971: 3). The rich fauna of the region was catalogued into 2864 species and (by Guimarães’ own admission) an over-split 944 genera.

There have been to date only two major attempts to correct the generic imbalance that has impeded study of New World Tachinidae, both by Donald Montgomery Wood. The first was a conspectus of the Blondeliini of North and Central America and the West Indies (Wood 1985). Although this study excluded South American Blondeliini, it nevertheless reduced the number of valid genera from about 230 to 55. Many of the genera sunk into synonymy were Townsend’s but there were also many described by Reinhard, Thompson, Curran and others. The second work to reduce the number of New World genera was Wood’s (1987) Tachinidae chapter in *Manual of Nearctic Diptera*. The nomenclatural changes in this work, including almost 200 new generic synonyms, were later enumerated by O’Hara and Wood (1998).

Wood (1987) also successfully bridged the gap between the generic classifications of the Nearctic and Palaearctic regions created by Townsend some decades earlier. This was accomplished partly by reducing the number of genera but also by assessing genera from a Holarctic perspective. The catalogue by O’Hara and Wood (2004) further united the classifications of Nearctic and Palaearctic Tachinidae. The catalogue by Guimarães (1971) has not been updated and the 800+ genera currently recognized in America south of the United States will not be easily converted into a modern classification. A careful study of the name-bearing types of the type species of many of these genera will be necessary before a better classification can be constructed for Neotropical Tachinidae.

### The European influence

The Europeans of the early 1900s continued to build on the discoveries of Girschner and others at the same time that Townsend in the New World was pursuing his own course of investigations that would culminate in his *Manual of Myiology*. Joseph Villeneuve de Janti, a medical doctor by profession (like Robineau-Desvoidy), emerged as an early specialist on the Tachinidae and published actively on the family from 1900 until his death in 1944. He wrote an influential paper in 1924 reviewing earlier works on chaetotaxy and detailing his own views on characters useful for understanding the evolution of the “Myodaires supérieurs”. This group comprised the “Tachinaires” (Tachinidae *sensu* present-day Oestroidea) and “Anthomyaires” (Anthomyiidae *sensu* present-day Muscoidea). Within Villeneuve’s Tachinidae were Calliphorinae, Sarcophaginae, Dexiinae, Rhinophorinae, Phasiinae, and Tachininae. Particularly noteworthy and progressive was the division of the Tachininae into two groups, Eutachininae and Protachininae, of which the former was considered more evolved than the latter. As a rough approximation, the two correspond to present-day Exoristinae and Tachininae, respectively.

Villeneuve was well respected by contemporaries for his expertise in Tachinidae and willingly shared his knowledge with others. As noted by Wainwright (1928: 141), Villeneuve:

“has contributed largely towards the reduction to something like order of our knowledge of these insects. Possibly the full value of his services to science may never be appreciated, because so many of the fruits of his labours have been given to the world by other workers, whom he has unselfishly and ungrudgingly assisted”.

The discovery by Malloch (1923) that the Tachinidae and Dexiidae can be distinguished from Sarcophagidae, Calliphoridae and Muscidae by an enlarged “metanotum” (subscutellum) was a highly significant development in the classification and differentiation of these flies. It was likely this discovery that led Villeneuve (1933) to revise his earlier classification and divide the “Tachinaires” into three groups:

1) Tachinidae with Phasiinae, Dexiinae and Tachininae,

2) Sarcophagidae with Miltogramminae, Sarcophaginae and Calliphorinae, and

3) Rhinophoridae, a small group of isopod parasitoids.

Villeneuve (1933) treated the Eutachininae and Protachininae of the Tachininae at length.

Villeneuve was a mentor and friend of Louis Paul Mesnil, who was 36 years his junior (Mesnil 1950). It was originally Villeneuve who was invited by Lindner to author the Tachinidae volumes of his ambitious *Die Fliegen der palaearktischen Region* (hereafter *FPR*). However, as the project drew closer Villeneuve realized that the talented and younger Mesnil was a better choice to take on this demanding and long-term task (Herting 1987).

Mesnil was an avid student of Tachinidae. He demonstrated his enthusiasm and insight early by publishing, as one of his first works on the group, a lengthy treatise entitled *Essai sur les Tachinaires* (Mesnil 1939). He began the *Essai* by reviewing and critiquing the classifications of his more illustrious predecessors: Robineau-Desvoidy, Macquart, Meigen, Rondani, Brauer and Bergenstamm, Pandellé, and Girschner. In proposing a new classification, Mesnil (1939) drew special inspiration from the works of Robineau-Desvoidy and Villeneuve, and like Brauer and Bergenstamm, started by grouping together related genera and building the classification “depuis la base vers le sommet” [“from the base to the summit”] (p. 20).

Mesnil (1939) restricted the term Tachinaires to the family Larvaevoridae^3^ (i.e., Tachinidae). The main diagnostic feature of the family was the well-developed “postscutellum” (subscutellum), as previously implied in Villeneuve’s (1933) classification and explicitly adopted by Curran (1934, as “metanotum”). Mesnil relegated the Rhinophorinae and Sarcophaginae to the Calliphoridae and subdivided the Larvaevoridae into six subfamilies: Salmaciinae^4^, Phorocerinae, Larvaevorinae, Ameniinae, Dexiinae and Phasiinae (including Oestrini). These were keyed and characterized and most were further subdivided.

The Phorocerinae of Mesnil (1939) consisted of tachinids possessing a haired prosternum and a small “prealar” (postsutural supraalar) seta. Included within the Phorocerinae were three tribes: Phorocerini, Blondeliini, and Crocutini^5^. The Phorocerini, with vein M (as “4^e^”) having an angular bend and a shadow fold, and the Blondeliini, with vein M having a rounded bend and no shadow fold, and both possessing divergent subapical scutellar setae (convergent in Crocutini), have continued to the present virtually unchanged in their characterization (Wood 1972, 1985). The Phorocerini have since become known as the Exoristini.

Mesnil began publishing *FPR* instalments a few years after his *Essai*. The goal was to treat all of the Palaearctic Tachinidae to species level but the task proved too great for him alone. After 35 years and some 1500 pages of text, the Larvaevorinae (present-day Exoristinae and Tachininae) were completed (Mesnil 1944–1975) along with one instalment on the Dexiinae (Mesnil 1980). Herting planned to publish on the remainder of the Dexiinae and all of the Phasiinae but only one instalment on the latter was published (Herting 1983).

Mesnil’s (1944) first instalment for *FPR* began, as did his *Essai*, with general remarks about previous workers and their classifications. Mesnil (1944: 2) made these observations about the generic concepts of other workers:

“Oft auch haben sie alte künstliche Gattungen aufrechterhalten, deren Umfang jedes Maß überschreitet und deren Heterogenität offenkundig ist; können sie doch sogar Arten verschiedener Tribus enthalten.

So lassen sich die meisten neuzeitlichen Dipterologen, da sie die wahren Merkmale der Tachinen zu wenig berücksichtigt haben, nach zwei Richtungen gruppieren: die einen unterteilen die Gattungen bis ins Unendliche und machen so fast alle monospezifisch (T. Townsend), die andern vereinigen zahlreiche Gattungen zu einem Ganzen und gelangen so zu monströsen Zusammenfassungen (Curran).”

[“Often, they have maintained old artificial genera whose scope exceeds all bounds and whose heterogeneity is obvious; even though they may contain species of different tribes.

Since most modern dipterists have not taken the true characteristics of tachinids into account, they can be grouped in two directions: some subdivide the genera into infinity and thus make almost all of them monospecific (T. Townsend), others unite numerous genera into a whole and arrive at monstrous compilations (Curran)”.]

Lindner (1933) established the classification of the Diptera that would be followed in *FPR* six years before Mesnil’s (1939) *Essai*. This constrained Mesnil (1944) into keeping Larvaevoridae in the older and broader sense of present-day Oestroidea instead of in the restricted sense of present-day Tachinidae. Recognized within Larvaevoridae were subfamilies Larvaevorinae (with tribes Salmaciini, Phorocerini and Larvaevorini), Dexiinae and Phasiinae. Mesnil’s (1939) Oestrini (then placed in Phasiinae) became the “Gastrophilinae” (Gasterophilinae), Oestrinae and “Hypoderminae” (Hypodermatinae) of Lindner (1933). It is clear that this higher classification did not appeal to Mesnil. To him, the true definition of the Larvaevoridae was undeniable (“unbestreitbare”) and based on the enlarged subscutellum and parasitic habits of the family (Mesnil 1944). His only recourse was to chart the classification he would have followed had he been permitted to do so (numbers in parentheses refer to Lindner’s numbering system for families) (Mesnil 1944: 20):

I Haplostomata Frey

II Thecostomata Frey

A Muscidae (63)

B Calliphoridae

a Calliphorinae (64i)

b Hypoderminae (64b)

c Sarcophaginae (64h)

d Rhinophorinae (64e)

C Larvaevoridae

a Phasiinae (incl. Oestrini) (64c)

b Dexiinae, Ameniinae (64f)

c Larvaevorinae (64g)

The Lindner series was published in small instalments (“Lieferungen”), the length of each being determined by the number of printed signatures used per instalment. Frequently an instalment would end in the middle of a description or in the middle of a key. This may have been cost-effective for the publisher but created havoc nomenclaturally. New generic names, for example, were often *nomina nuda* in one instalment and not made available until years later in another instalment. A great number of such nomenclatural issues as they pertain to the Tachinidae were dealt with by O’Hara (1996), Evenhuis and O’Hara (2008), and Evenhuis et al. (2008).

Mesnil’s *FPR* instalments by definition dealt primarily with the Palaearctic fauna but incorporated information on the taxa of other regions, except for the nearly impenetrable taxa of Neotropical Tachinidae. The result, in concert with a great many papers published by Mesnil outside *FPR*, was a classification for the bulk of the Tachinidae that could be hailed by contemporaries as a leap forward in the quest for a scheme reflecting the true relationships of the family. The suprageneric classification of Townsend (1934–1942) was largely ignored by Europeans who were making progress through their own investigations.

The first of Mesnil’s (1944) instalments in *FPR* gave only a glimpse of the classification that would follow. The Ameniinae were transferred to the Calliphoridae and kept as a subfamily, although the family itself is not currently considered monophyletic (e.g., Rognes 1997, Kutty et al. 2010). Mesnil’s (1944) three tribes of Larvaevorinae were split over the duration of *FPR* into a number of subtribes: nine in Salmaciini, six in Phorocerini and over 40 in Larvaevorini. The Larvaevorini were revisited by Mesnil (1966) and reclassified as Tachinini *s. str.* and Voriini. All of the subtribes of Mesnil (1944–1975) are now generally tribes and tribe Larvaevorini is present-day Tachininae. Many of the tribes continue to this day virtually unchanged whereas a few have undergone dramatic restructuring in the light of subsequent discoveries. The most significant changes resulted from research on the female postabdomen by Herting (1957) and male genitalia by Verbeke (1962a).

Benno Herting began his career on Tachinidae much the same way as did Mesnil (and even Robineau-Desvoidy) with an early publication based on original and extensive research (Herting 1957). It was a study of the female postabdomen and was based on the examination of about 500 species of calyptrate flies. Information about eggs and first instar larvae were taken into account but unlike Townsend’s studies the focus was more on the morphology of the terminal segments of the postabdomen than on the internal reproductive system. Herting (1957) used his findings to characterize the structural features of the female postabdomen throughout the families, subfamilies and lower groups of the Calyptratae. He tried to interpret these findings in a phylogenetic context and to adjust the classification accordingly.

Five subfamilies of the Tachinidae were recognized by Herting (1957): Echinomyiinae^6^, Dexiinae, Phasiinae, Ocypterinae^7^, and Eutachininae. At a gross level, Echinomyiinae corresponded to the Protachininae of Villeneuve (1924, 1933) and to the Larvaevorinae of Mesnil (1939; and later, Larvaevorini of Mesnil 1966–1975); Ocypterinae was formerly treated within Phasiinae by both Villeneuve (1924, 1933) and Mesnil (1939); and Eutachininae was proposed by Villeneuve (1924) and corresponded to the Salmaciinae (-ini) and Phorocerinae (-ini) of Mesnil (1939, 1944). Herting (1957) treated the Oestridae as a separate family.

Herting (1957) followed Villeneuve (1924, 1933) in using the subfamily name Eutachininae in his classification. He subdivided this subfamily into the Goniini and Eutachinini. He could not find reliable characters in the female postabdomen to separate these tribes and therefore chose to organize his discussion according to the reproductive habits of the species. Oviparous species were placed in the Eutachinini and distributed mostly between the *Winthemia* Robineau-Desvoidy group and *Eutachina*^8^ Brauer and Bergenstamm group. These were essentially the Winthemiina and Phorocerina that Mesnil (1944) had placed in tribes Salmaciini and Phorocerini, respectively. Ovolarviparous species grouped by Mesnil (1944) in the Blondeliina (tribe Phorocerini) were also assigned to the Eutachinini. The ovolarviparous *Siphona* Meigen group (Siphonina, tribe Phorocerini, of Mesnil 1944) was more clearly defined but its placement in Eutachinini or Goniini was not discussed. Similarly, the “*Ethylla*” (*Ethilla*) Robineau-Desvoidy group was included in Eutachininae but its further placement was not discussed. No members of the Acemyina (tribe Phorocerini) of Mesnil (1944) were studied by Herting (1957).

The composition of Herting’s (1957) Goniini consisted of species with two reproductive modes. One is quite specialized and involves the production of tiny (microtype) eggs that females oviposit on the food plants of hosts. These eggs hatch only after ingestion by a potential host. This sort of egg and the biology associated with it were already well known as a result of earlier studies (e.g., Sasaki 1887, Townsend 1908, 1911, Pantel 1910^9^). The rest of Herting’s (1957) Goniini were mostly ovolarviparous species with a few oviparous species. This broad concept of the Goniini was essentially the Salmaciinae (-ini) of Mesnil (1939, 1944) without Ethyllina and Winthemiina.

Herting (1957) introduced an important change to the placement of the Voriini. The members of this tribe had been included in the Protachininae of Villeneuve (1924, 1933) and the nearly equivalent Larvaevorinae of Mesnil (1939). Herting (1957) placed the tribe in the Dexiinae, bringing to three the number of Palaearctic tribes recognized in the subfamily: Dexiini, Voriini and Dufouriini. This move was supported by female postabdominal characters and by features of the male genitalia communicated to Herting by Verbeke (see below).

Mesnil (1956–1965) published on the Phorocerini in *FPR* over a ten-year period. He subdivided the tribe into subtribes Phorocerina, Blondeliina, Atylomyina, Neominthoina, Acemyina, and Siphonina, describing all the Palaearctic species and working in the same meticulous way that he had earlier for the Salmaciini (Mesnil 1944–1956). He had already revised the Old World Phorocerina (as Phorocerini) in a separate publication (Mesnil 1946) that he had probably begun before starting *FPR*. Mesnil (1956–1965) was halfway through the Phorocerini when Herting published his next great work on the Tachinidae, a monograph on the biology of the West Palaearctic species (Herting 1960). This work had a different focus from his earlier study but included a hierarchical arrangement of taxa that the former work had lacked. A clear classification was in evidence and although it was congruent in many respects with Mesnil’s it differed from it in some significant ways. Herting (1960) proposed a major restructuring of Mesnil’s Salmaciini (Mesnil 1944–1956) and Phorocerini (Mesnil 1956–1965). Both were united to form the Exoristinae^10^, consisting of a broadly defined Goniini (see above), Ethillini (Mesnil’s Ethyllina and Atylomyina), and the following tribes that corresponded to Mesnil’s remaining subtribes (except for the mixed and non-Palaearctic Neominthoina): Winthemiini, Exoristini (Mesnil’s Phorocerina), Blondeliini, Acemyiini, and Siphonini.

Herting’s (1960) Echinomyiinae included just three tribes: Echinomyiini, Leskiini and Microphthalmini. This work was published after Mesnil (1939) but before the *FPR* instalments on the same group (Mesnil 1966–1975, as “Larvaevorini oder Tachinini”). Mesnil (1939) had treated this group as the Larvaevorinae and noted that it was very close to Villeneuve’s (1933) Protachininae except for the exclusion of section *Winthemia* (placed by Mesnil in Salmaciinae [= Villeneuve’s Eutachininae], as Winthemiini). Mesnil’s (1939) Larvaevorinae had consisted of eight tribes^11^: Campylochaetini, Athryciini, Larvaevorini, Rhamphinini, Leskiini, Minthoini, Thelairini, and Macquartiini. This heterogeneous assemblage was considerably altered by Herting (1960): Larvaevorini and part of Macquartiini were placed in Echinomyiini; Campylochaetini, Athryciini, Thelairini and part of Macquartiini (i.e., the Phyllomyina) were moved to Voriini in the Dexiinae; Minthoini were included in Leskiini; and Rhamphinini were not treated but were later placed in Voriini by Herting (1984). The Microphthalmini of Herting (1960) were moved to the Tachininae from Mesnil’s (1939) section Dexiosomina (Dexiini, Dexiinae).

At the same time that Mesnil (1956–1965) was working through the Phorocerini using external characters and Herting (1957, 1960) was studying the female postabdomen, Jean Verbeke (1962a, 1962b^12^, ^1963^) was investigating tachinid male genitalia. Verbeke was communicating some of his findings to Herting before publishing them himself, thus contributing at least to Herting’s concept of the Dexiinae (see above). Verbeke (op. cit.) recognized within the complexity of the male genitalia a few general “types” associated with three structures. Firstly, the connection between the basiphallus and distiphallus is either “direct and non-mobile” (type I) or “indirect and mobile” (type II). Secondly, the distiphallus either lacks (POS [= *Phasia*, *Ocyptera*, *Strongygaster*] type) or possesses (DEG [= *Dexia*, *Echinomyia*, *Gonia*] type) longitudinal ventral microstructures. Thirdly, the “posterior paramere” (pregonite) has three types: type A, lobe-like and sensorial; type B, intermediate; and type C, strap-like and connective. These structural types do not form unique combinations and Verbeke (1963: 4) understood that “this repeated appearance of similar structures in different groups implicates a parallelism between the male genitalia of these groups”. Verbeke (1962a) concluded that the Tachinidae were best divided into six subfamilies: Phasiinae were characterized on the basis of a POS type distiphallus, whereas other Tachinidae have a DEG type distiphallus; Echinomyiinae (i.e., Tachininae) and Eutachininae (i.e., Exoristinae) have a type I connection between basiphallus and distiphallus; Dexiinae and Voriinae have a type II connection between basiphallus and distiphallus; and Dufouriinae with tribes Macquartiini and Dufourini, the former with a type I connection between the basiphallus and distiphallus and the latter with a type II connection but both tribes having a pregonite of type B. The subfamily Dufouriinae was clearly one of convenience and was not thought to be monophyletic. Verbeke (1963: 3) noted:

“Many other characters prove the intermediate situation of both tribes [intermediate between Dexiinae-Voriinae and Echinomyiinae-Eutachininae, see illustration in Verbeke (1962a: 147)] and for this reason we fused them into a new subfamily”.

Herting (1957, 1960) was aware of Verbeke’s studies on the male genitalia in advance of the publications on this subject (Verbeke 1962a, 1963) and was also familiar with the pioneering work on male genitalia by Rubtzov (1951). Herting (1957) discovered that features in the female postabdomen—and corroborated by evidence from the male postabdomen—supported a new concept of the Dexiinae. The Dexiini, Voriini and Dufouriini were brought together to form the Dexiinae. Although this classification differed from the one proposed later by Verbeke (1962a, 1963), it can be seen that Verbeke’s type II phallus and type C pregonite accurately defines Herting’s (1957, 1960) Dexiinae. This understanding of the subfamily continues to this day (e.g., Herting 1984, Tschorsnig 1985, Wood 1987, Tschorsnig and Richter 1998, O’Hara and Wood 2004, Cerretti 2010). Verbeke’s Macquartini, the other half of his Dufouriinae, was placed by Herting (op. cit.) in the Echinomyiinae but not retained as a tribe.

Mesnil (1966–1975) next published a series of instalments in *FPR* on the Larvaevorini, or Tachinini *s. lat.* In the first instalment, Mesnil (1966) introduced some changes to his earlier classification of the Larvaevorinae (i.e., Tachinidae). The classification proposed consisted of six tribes (equivalent to subfamilies of other authors): Phasiini, Exoristini, Goniini, Dexiini, Voriini, and Tachinini *s. str.* (see chart, Mesnil 1966: 882). The first three were characterized as producing planoconvex eggs and the last three as producing membranous eggs. Herting (1966) also noted this distinction in egg type between what he considered the two lineages of Tachinidae. Mesnil (1966) recognized the Phasiini as distinct based on the POS-type distiphallus of Verbeke (1962a) and the characteristic female postabdomen of Herting (1957). An unusual group that defies easy placement to this day, the Eutherina, were placed in the Voriinae by Verbeke (1962a) (based on male genitalia) and in the Phasiinae (-ini) by both Herting (1966) and Mesnil (1966) (based on egg type).

Mesnil (1966) was further influenced by Herting (1957) and Verbeke (1962a) to remove the voriines from the Larvaevorinae (-ini) of Mesnil (1939, 1944) and place them next to the dexiines. He kept the groups separate as Voriini and Dexiini rather than place them in the Dexiinae as did Herting (1957). The Dufouriinae of Verbeke (1962a) were split along similar lines to Herting (1957, 1960) with the Dufourini moved to Voriini as Dufouriina and Macquartiini kept in Tachinini *s. str.* (as Macquartiina) following Mesnil (1939). The original Dufouriina of Mesnil (1939) was a mixed group placed in Phasiini of Phasiinae and included such aberrant genera as *Graphogaster* Rondani and *Rondaniooestrus* Villeneuve in addition to *Dufouria* Robineau-Desvoidy and other typical dufouriines. Mesnil (1966) treated a more restricted Dufouriina in Voriini, placed *Graphogaster* in the small subtribe Graphogastrina in Tachinini *s. str.*, and recognized *Rondaniooestrus* as sole member of Rondaniooestrina in Phasiini.

The Tachinini *s. str.* of Mesnil (1966) were split among about 30 subtribes. This tribe was equivalent to Mesnil’s (1939) Larvaevorinae and its eight tribes except for the removal of the voriines. In revising the earlier classification of Mesnil (1939) for *FPR*, Mesnil (1966) reduced his former tribes to subtribes and raised some former sections to tribes (especially among the Larvaevorini and Macquartiini of Mesnil 1939). This classification bears some resemblance to the groupings of Brauer and Bergenstamm (1889–1895) and Townsend (1934–1942) and reflected the uncertainty inherent in attempting to classify this heterogeneous and likely polyphyletic assemblage.

The Dexiosomina, treated in Dexiini of Dexiinae by Mesnil (1939), became part of Mesnil’s (1966–1975) Microphthalmina in Tachinini *s. str.*

Over 30 years elapsed between Mesnil’s (1944–1975) first and last *FPR* instalments on the Larvaevorinae. Mesnil (1975a, 1975b) included an Addenda and Corrigenda at the end of the Larvaevorini section in which he made corrections to earlier mistakes, added notes, and revised certain groups. His most significant change concerned the Goniinae (Salmaciini of Mesnil 1944–1956; i.e., present-day Exoristinae). This group had been based on external characters and needed revision to conform to the reproductive types discussed by Herting (1957, 1960). Mesnil (1975a: 1374) concluded:

“Nach Untersuchungen, die besonders durch B. Herting 1957 … über die Anatomie des Postabdomens der mikrooviparen Weibchen durchgeführt wurden, ist es möglich, die Gattungen der Goniinae in 2 Triben zu ordnen: die Goniini Rob.-Desv. (1830) mit mikrotypen Eiern und die Eryciini Rob.-Desv. (1830), die ovolarvipar oder ovipar sind.” [“According to studies that have been carried out especially by B. Herting 1957 … on the anatomy of the postabdomen of microoviparous females, it is possible to arrange the genera of Goniinae into two tribes: the Goniini Rob.-Desv. (1830) with microtype eggs and Eryciini Rob.-Desv. (1830), which are ovolarviparous or oviparous.”]

Goniini (*s. str.*) + Eryciini of Mesnil (1975a, 1975b) corresponded to Goniini (*s. lat.*) + Winthemiina + Ethillina of Herting (1960). Mesnil’s restriction of the Goniini to microovolarviparous tachinids was a key development in the classification of the Exoristinae. Herting (1984) would later remove the Winthemiina and Ethillina from Eryciini and treat them as tribes of Exoristinae, thereby creating a concept of Goniini *s. str.* + Eryciini equaling that of Herting’s (1960) Goniini.

The microovolarviparous tachinids had been recognized informally as a natural group within a broader Goniini since Herting’s (1957) study of the female postabdomen. A few years later Herting (1960) again grouped these tachinids as the “Mikroovipare Arten” within his broadly defined Goniini. Herting was known to be in favour of classifying the Goniini in a more restricted sense even before this was proposed by Mesnil (1975a). Very likely the idea was more his than Mesnil’s, although the two colleagues surely discussed the issue and may have influenced each other in how best to classify these tachinids. What is known is that Herting corresponded with others about his thoughts on this suprageneric complex prior to Mesnil (1975a) publishing on it. This is evident in Crosskey’s (1973b: 77) comments on the tribal classification he was adopting for Australian Goniinae (i.e., Exoristinae):

“Herting (personal communication) considers that the multifarious genera of the Goniini-Carceliini-Sturmiini-Eryciini complex should be aggregated into two tribes (for which the names Eryciini and Goniini would be nomenclaturally correct) according to whether they have an ovolarviparous or a microoviparous reproductive habit. Such a course has much to commend it insofar as it would probably reflect the real phylogeny more accurately than the present tribal system. But it is impossible to adopt such a system as yet for the Australian fauna, in which the reproductive habit of most of the genera remains unstudied.”

Thompson (1963), based on his own study of innumerable dissections, also recognized the microovolarviparous tachinids as a distinct group and devoted a separate part of *Tachinids of Trinidad* to the “goniines with microtype eggs”. Thompson (1963: 258) noted: “In the classification of Townsend, species producing microtype eggs are scattered through at least 14 tribes: Eriothrixini, Compsilurini, Phoroceratini, Phorinini, Actiini, Hyperecteinini, Frontinini, Goniini, Belvosiini, Harrisiini, Sturmiini, Lydellini, Phrynoini and Trypherini.”

Sabrosky and Arnaud (1965) (see also above) were caught between the Townsend legacy of New World tachinid taxonomy and the rapidly evolving views on tachinid relationships and classification of the European specialists Mesnil, Herting and Verbeke. Sabrosky and Arnaud (1965) recognized both the Goniini and Eryciini but neither tribe corresponded very closely to the Goniini and Eryciini later defined by Mesnil (1975a, 1975b).

There was no Palaearctic catalogue of Tachinidae published between those of Bezzi and Stein (1907) and Herting (1984). Authors in the Old World wishing to treat regional faunas during this period were given overviews of emerging classifications first by Villeneuve (1924, 1933) and then by Mesnil (1939, 1944–1975), with contributions in particular from Herting (1957, 1960) and Verbeke (1962a). Villeneuve was acknowledged as a significant influence in the regional treatments of Stein (1924), Lundbeck (1927) and Wainwright (1928). As noted above in a quote from Wainwright (1928), Villeneuve’s personal assistance to contemporary dipterists was as valuable a contribution to science as were his publications.

Before the Second World War, tachinid specimens from Africa were routinely sent to the Imperial (later Commonwealth) Institute of Entomology in London for identification, but in practise they were identified by Villeneuve in France. This changed when the war severed relations with Villeneuve and the task of identifying Tachinidae fell to the recently hired dipterist, Fritz Isidor van Emden. Thus began van Emden’s foray into the Tachinidae that resulted in his valuable contributions on the faunas of the Afrotropical (as “Ethiopian”) Region (van Emden 1945, 1947, 1960 [the last posthumously]) and British Isles (van Emden 1954). In choosing a classification to follow, van Emden (1954: 7) noted:

“a sound classification has only recently been suggested by Villeneuve (1924, 1933) and worked out by Mesnil (1939, 1944). Being of such recent date, this ingenious classification has not so far been checked and applied to the whole of the family.”

Van Emden was slightly too early to take advantage of the progress to come during the 1960s through the efforts of Mesnil, Herting and Verbeke. Van Emden had planned to prepare keys to the whole of the Afrotropical Tachinidae but died before the third part was published (van Emden 1960) and before the last and largest part (on Exoristinae, as “Goniinae”) could be started.

Dugdale (1969) was more fortunate in being able to consider the works of Herting (1957, 1960), Verbeke (1962a), and Dupuis (1963) along with the recently revised classification of Mesnil (1966) in his treatment of New Zealand Tachinidae. Dupuis (1963) had concerned himself exclusively with the Phasiinae and his classification of the subfamily differed from that of Verbeke’s principally in the exclusion of the Strongygasterini and Rondaniooestrini. Despite Dugdale’s (1969) detailed review of recent advances, the New Zealand fauna is a small and isolated one and the affinities of some of its taxa were not resolved by Dugdale and remain uncertain to this day.

Roger Ward Crosskey became the next dipterist with the Commonwealth Institute of Entomology after the death of van Emden. His would be a remarkable tenure, single-handedly producing a revision of the Rutiliini (a tribe of Dexiinae confined to the Oriental and Australasian regions, Crosskey 1973a), conspecti on the Tachinidae of Australia (Crosskey 1973b) and the Oriental Region (Crosskey 1976), a catalogue of the Afrotropical^13^ Tachinidae (Crosskey 1980b), and keys to the tachinid genera of tropical and southern Africa (Crosskey 1984). Additionally, Crosskey later assisted with the preparation of a catalogue of the Tachinidae of the Australasian and Oceanian regions (Cantrell and Crosskey 1989). These resources offered a wealth of information on the names, classification, identification and hosts of Old World non-Palaearctic Tachinidae. The function of these works, however, was not to investigate and further illuminate the phylogenetic relationships of the Tachinidae. Perhaps for this reason and for the sake of consistency, the classificatory scheme adopted for the earliest conspectus was carried through with little change to the final catalogue, despite advances in tachinid systematics in the interim.

The classifications of Crosskey (1973b, 1976, 1980b) and Cantrell and Crosskey (1989) are very nearly the same and are best compared to the overview of tachinid classification given by Mesnil (1966) and, with respect to the Goniini–Eryciini, Mesnil (1975a). The classification in these works differed from that of Mesnil most significantly in the following respects^14^:

1) Tachininae included, in addition to the Tachininae *sensu* Herting (1984), most of Mesnil’s (1966) Voriini as tribes Campylochetini, Parerigonini, Phyllomyini, Thelairini, Voriini, and Wagneriini. Mesnil’s (1966) voriine subtribe Dexiomimopsina was included in Leskiini (later, *Dexiomimops* Townsend was treated in Voriini of Dexiinae by Herting 1984 and Shima 1987).

2) The “Goniini-Carceliini-Sturmiini-Eryciini complex” of Goniinae (i.e., Exoristinae) was not divided into Goniini and Eryciini according to egg type as advocated by Herting (see quote above from Crosskey 1973b) and Mesnil (1975a). Crosskey (1973b) gave two practical reasons for this: the reproductive habits of most of the genera involved were unknown and separating the redefined Goniini and Eryciini in a key on the basis of external morphology would not be possible even if egg type of each genus was known.

3) Dufouriinae were recognized as a subfamily with tribes Dufouriini and Imitomyiini; Mesnil (1966) had treated the former as a subtribe of Voriini and the latter as a subtribe of Phasiini.

4) Doleschallini were recognized as a tribe of Dexiinae; Mesnil (1966) had treated the single Oriental/Australasian genus *Doleschalla* Walker in the Doleschallina of Voriini^15^.

5) Oxyphyllomyiini were recognized as a tribe of Tachininae; Mesnil (1966) had treated the single Oriental genus *Oxyphyllomyia* Villeneuve in the Oxyphyllomyiina of Voriini. Later, Shima (1983) transferred *Oxyphyllomyia* to Leskiini.

6) Thelairini of Tachininae included Mesnil’s (1966) Zambesina of Exoristini (see discussion, Crosskey 1973b: 75).

7) Palpostomatini and Glaurocarini were recognized as tribes of Tachininae; Mesnil (1966) had treated both as subtribes of Exoristini.

8) Neaerini and Siphonini were recognized as tribes of Exoristinae; Mesnil (1966) had treated both as subtribes of Tachinini.

9) Rondaniooestrini were placed in Tachininae; Mesnil (1966) had treated the Rondaniooestrina as a subtribe of Phasiini.

### The Modern Era

Nearly 25 years after writing about the biology of the West Palaearctic Tachinidae (Herting 1960) and over 75 years after the Palaearctic Tachinidae were last catalogued (Bezzi and Stein 1907), Herting (1984) published a long-awaited *Catalogue of Palearctic Tachinidae*. Much had changed since the former catalogue, both in terms of the suprageneric classification and number of genera and species. The tachinid fauna of the Palaearctic Region was the most intensively studied of all regional faunas and an up-to-date catalogue was an invaluable resource. Mesnil’s classification had evolved significantly over the years since publication of *Essai sur les Tachinaires* in 1939 but the changes had taken place in stages and must not have been easy for a non-specialist to follow. Herting had introduced changes too, some accepted by Mesnil and others not. Coincidently, Herting’s (1984) catalogue came out at the end of Mesnil’s long career and there have not been any sweeping changes to tachinid classification since. What has changed will be discussed further on. Herting (1984: 2) compared his classification to that of Herting (1960):

“The subdivision into four subfamilies is the same, only the name Echinomyiinae had to be changed into Tachininae. Some alterations have been made on the tribal level: The tribe Goniini is now restricted to the microoviparous forms, whereas the oviparous and ovolarviparous genera are assembled in a separate tribe, Eryciini. In the subfamily Tachininae, the number of tribes has been moderately increased, but not all the divisions made by Mesnil (1966b) in Lindner 64g: 885–896, have been accepted. The Siphonini are transferred from the Exoristinae to the Tachininae, where they are certainly better placed.”

The classification of Herting (1984) differed from that of Mesnil (1966, 1975a) primarily in the following respects:

1) Winthemiini and Ethillini were recognized as tribes of Exoristinae; Mesnil (1975a) had included them in Eryciini, the former as Winthemiina and the latter as the three subtribes Ethillina, Phorocerosomina, and Atylomyina.

2) Dufouriini were recognized as a tribe of Dexiinae; Mesnil (1966) had treated Dufouriina as a subtribe of Voriini.

3) Voriini were recognized, without subtribes, alongside Dexiini and Dufouriini as one of three Palaearctic tribes in Dexiinae. Mesnil (1966) had treated his Voriini on the same level as the Voriinae of Verbeke (1962a) with 17 subtribes (see above for the treatment of Mesnil’s Voriini in Tachininae by Crosskey).

4) Tribes of Tachininae were significantly reduced from the subtribes of Tachinini of Mesnil (1966), although there was a sizable increase over the three tribes formerly recognized by Herting (1960). This increase over Herting (1960) was due primarily to a finer splitting of Echinomyiini and the separation of Minthoini from Leskiini.

Following closely after Herting’s (1984) catalogue was a comprehensive and insightful study of the male postabdomen by Tschorsnig (1985), Herting’s student and later his successor in Stuttgart. Tschorsnig took a comparative approach, describing the structures comprising the male postabdomen, detailing variation throughout the family, and discussing at the end of each taxonomic group the evidence regarding affinities. The work was less focused on the phallus and the pre- and postgonites than that of Verbeke (1962a) and arrived at some different conclusions. For example, the Phasiinae were considered monophyletic based on the structure of the hypandrium rather than on Verbeke’s POS type distiphallus, and the Dexiinae of Herting (1984) and not Verbeke (1962a) were considered monophyletic based on Verbeke’s type II phallus and type C pregonite. Although Tschorsnig’s study was phylogenetic in nature it did not include a cladogram of inferred relationships. The author may have considered the subject too complex and uncertain to condense into a single cladogram and may have preferred instead to present information about possible relationships in a narrative format.

Cantrell (1988) also conducted a comparative study, this one on the postabdomen of both sexes of Australian Tachinidae with descriptions of first instars and puparia. It was based on a thesis that was presumably completed prior to the publication of Tschorsnig (1985) because this work was not cited. The study provided a good overview as well as notes about each tribe of Australian Tachinidae.

Herting’s (1984) catalogue has been particularly influential to modern tachinidology because it summarized the current state of knowledge after a long period of change and has been followed subsequently by a period of relative stability. There have been highly significant works on Tachinidae published since 1984 but no revolutionary ideas have emerged about higher level relationships and classification. This is not to say that Herting’s classification is a true reflection of tachinid phylogeny, but rather it has changed little because the large groups that are least likely to be monophyletic (e.g., Eryciini, Tachininae, Voriini) have remained too little understood to permit their reclassification along phylogenetic lines.

Some major regional treatments and larger taxonomic works since Herting (1984) are reviewed below. There is still uncertainty about the proper placement of certain taxa among some of these works and in comparison with the major works during Mesnil’s era. These differences mostly concern smaller taxonomic units, often genera, and rather than discuss them below they are listed in Table 1.

**Table 1.** The varied taxonomic placements of certain taxa of the Tachinidae by different authors are shown. Square brackets are used to indicate that a family-group name based on the taxon in question is given in the work but the taxon itself is not named in the work.<br/>

**Taxon / Authors**	***Acemya* Rob.-Des.**	***Campylocheta* Rondani**	***Dufouria* Rob.-Des.**	***Euthera* Loew**	***Imitomyia* Townsend**	***Microphthalma* Macquart**	***Oxyphyllomyia* Villeneuve**	***Palpostoma* Rob.-Des.**	***Rondaniooestrus* Villeneuve**	***Strongygaster* Macquart**	***Thelaira* Rob.-Des.**	**Goniini**	**Neaerini**	**Siphonini**	**Voriini**
Herting (1960)	Exoristinae, Acemyiini	Dexiinae, Voriini	Dexiinae, Dufouriini	—	—	Tachininae, Microphthalmini	—	—	—	Phasiinae, Strongygastrini	Dexiinae, Voriini	Goniini *s. lat.*^6^	Echinomyiinae, Echinomyiini	Exoristinae, Siphonini	Dexiinae, Voriini
Verbeke (1962a, 1963)	Eutachininae^1^, Acemyini	Voriinae, *Campylocheta* group	Dufouriinae, Dufouriini	Voriinae, *Euthera* group	Dufouriinae, Macquartiini^2^	Echinomyiinae^1^, *Microphthalma* group	—	Dufouriinae, Macquartiini^2^	Phasiinae, Strongygastrini^7^	Phasiinae, Strongygastrini^7^	Dexiinae, Thelairini	Goniini *s. lat.*^6^	Echinomyiinae^1^, *Gymnocheta* group	—	Voriinae
Sabrosky and Arnaud (1965)	Goniinae^3^, Acemyini	Tachininae, Campylochetini	—	Phasiinae, Eutherini	Phasiinae, Imitomyiini	Proseninae, Dexillini	—	[Phasiinae, Palpostomatini]	—	Phasiinae, Strongygastrini	Dexiinae, Thelairini	Goniini (restricted)^6^	Goniinae, Siphonini, Neaerina	Goniinae, Siphonini, Siphonina	Tachininae, Voriini
Mesnil (1966)^4^	Exoristini, Acemyina	Voriini, Campylochetina	Voriini, Dufouriina	Phasiini, Eutherina	Phasiini, Imitomyina	Tachinini, Microphthalmina	Voriini, Oxyphyllomyina	Exoristini, Palpostomatina	Phasiini, Rondaniooestrina	Phasiini, Strongygastrina	Voriini, Thelairina	Goniini *s. lat.*^6^	Tachinini, Neaerina	Tachinini, Siphonina	Voriini *s. lat.*
Guimarães (1971)	[Goniinae, Acemyini]	Tachininae, Campylochetini	—	Phasiinae, Eutherini	—	Proseninae, Dexillini	—	—	—	Phasiinae, Strongygastrini	Dexiinae, Thelairini	Goniini (restricted)^6^	[Goniinae, included in Siphonini]	Goniinae, Siphonini	Tachininae, Voriini
Crosskey (1973, 1976, 1980b, 1984)	Goniinae, Acemyini	Tachininae, Campylochetini	[Dufouriinae, Dufouriini]	Phasiinae, Eutherini	Dufouriinae, Imitomyiini	Tachininae, Microphthalmini	Tachininae, Oxyphyllomyiini	Tachininae, Palpostomatini	Tachininae, Rondaniooestrini	—	Tachininae, Thelairini	Goniini (restricted)^6^	Goniinae, Neaerini	Goniinae, Siphonini	Tachininae, Voriini
Herting (1984), Herting and Dely-Draskovits (1993)	Exoristinae, Acemyini	Dexiinae, Voriini	Dexiinae, Dufouriini	Phasiinae, Eutherini	—	Tachininae, Microphthalmini	—	—	—	Phasiinae, Strongygastrini	Dexiinae, Voriini	microtype Goniini	Tachininae, Neaerini	Tachininae, Siphonini	Dexiinae, Voriini
Cantrell and Crosskey (1989)	[Goniinae, Acemyini]	Tachininae, Campylochetini	—5	Phasiinae, Eutherini	—	Tachininae, Microphthalmini	—	Tachininae, Palpostomatini	—	Phasiinae, Strongygastrini	Dexiinae, Voriini	Goniini (restricted)^6^	Tachininae, Neaerini	Tachininae, Siphonini	Dexiinae, Voriini
Shima (1989)	—	—	—	Dexiinae	—	—	—	—	—	Phasiinae, Strongygastrini	—	microtype Goniini	—	—	Dexiinae, Voriini
Ziegler (1998)	Exoristinae, Acemyini	Dexiinae, Voriini	Dexiinae, Voriini	Phasiinae, Eutherini	—	Tachininae, Microphthalmini	—	Tachininae, Palpostomatini	—	Phasiinae, Strongygastrini	Dexiinae, Voriini	microtype Goniini	Tachininae, Neaerini	Tachininae, Siphonini	Dexiinae, Voriini
Richter (2004)	Exoristinae, Acemyini	Dexiinae, Voriini	Dexiinae, Dufouriini	Phasiinae, Eutherini	Phasiinae Imitomyiini	Tachininae, Microphthalmini	—	—		Phasiinae, Strongygastrini	Dexiinae, Voriini	microtype Goniini	Tachininae, Neaerini	Tachininae, Siphonini	Dexiinae, Voriini
O’Hara and Wood (2004)	Tachininae, Acemyini	Dexiinae, Campylochetini	Dexiinae, Dufouriini	Dexiinae, Eutherini	Dexiinae, Imitomyiini	Tachininae, Megaprosopini	—	Dexiinae, Palpostomatini	—	Phasiinae, Strongygastrini	Dexiinae, Voriini	microtype Goniini	Tachininae, Neaerini	Tachininae, Siphonini	Dexiinae, Voriini
O’Hara et al. (2009)	Tachininae, Acemyini	Dexiinae, Campylochetini	Dexiinae, Dufouriini	Dexiinae, Eutherini	Dexiinae, Imitomyiini	[Tachininae, Megaprosopini]	Tachininae, Leskiini	Tachininae, Palpostomatini	—	Tachininae, Strongygastrini	Dexiinae, Thelairini	microtype Goniini	Tachininae, Neaerini	Tachininae, Siphonini	Dexiinae, Voriini
Cerretti (2010)	Exoristinae, Acemyini	Dexiinae, Voriini	Dexiinae, Dufouriini	Dexiinae, Eutherini	[Dexiinae, Imitomyiini]	Tachininae, Megaprosopini	—	—	—	Tachininae, Strongygastrini	Dexiinae, Voriini	microtype Goniini	Tachininae, Neaerini	Tachininae, Siphonini	Dexiinae, Voriini

^1^ Eutachininae and Echinomyiinae were used in the sense of Exoristinae and Tachininae (or Larvaevorinae), respectively.

^2^ Verbeke (1962a) was often unclear when applying his findings to a classification. With respect to *Imitomyia* and *Palpostoma*, these were part of Macquartiini (also as ‘Macquartiines’) in Part 1 but were treated as Imitomyiini and Palpostomatini in Part 2, although in the latter they were presumably still in a subordinate relationship with Macquartiini of Part 1.

^3^ The name Goniinae was changed to Exoristinae when the latter was determined to have priority.

^4^ Mesnil (1966: 882) treated all Tachinidae as Tachininae and recognized six tribes, the equivalent of other author’s subfamilies.

^5^ Cantrell and Burwell (2010) recognized the Dufouriini as a tribe of Dexiinae.

^6^ “Goniini *s. lat.*” comprises both microtype and non-microtype taxa and “Goniini (restricted)” comprises only a portion of the microtype taxa.

^7^ Verbeke (1963) was not certain about the phylogenetic position of *Strongygaster* and *Rondaniooestrus* and suggested they might be “intermediaries” between Phasiinae and Dufouriinae.

Among the larger regional treatments of the 1980s were Cantrell’s (1984) study of Australian Phasiinae and Wood’s (1985) conspectus of the Blondeliini of North and Central America and the West Indies (the latter discussed above). The first modern key to the genera of Nearctic Tachinidae was published by Wood (1987) in *Manual of Nearctic Diptera*. The Siphonini of the world were revised at the generic level by O’Hara (1989). The Tachinidae of the Australasian and Oceanian regions were catalogued by Cantrell and Crosskey (1989), not only bringing Crosskey’s (1973b) conspectus of Australian Tachinidae up-to-date but cataloguing for the first time the non-Australian tachinids of the Australasian and Oceanian regions. Shima (1989) published a general paper on tachinids aimed at a Japanese audience; this work, unpretentious in nature, was remarkably detailed and presented the first cladogram of inferred relationships among the major (and controversial) tachinid lineages.

Other than the detailed study of the systematics of Australasian Dexiini by Barraclough (1992), the 1990s were dominated by European authors. Pape (1992) published on the phylogeny of the Tachinidae family-group, wherein the Tachinidae were inferred to form a monophyletic group (see also analysis by Pape and Arnaud 2001). Belshaw (1993) produced a handbook to the tachinids of the British Isles, replacing the earlier handbook by van Emden (1954). A new Palaearctic catalogue of the Tachinidae was published by Herting and Dely-Draskovits (1993) in the series *Catalogue of Palaearctic Diptera*, essentially reproducing the catalogue of Herting (1984) with corrected spellings to conform with nomenclatural rules and including long lists of *nomina dubia* not given in the earlier catalogue. Tschorsnig and Herting (1994) produced a valuable work on the identification, distribution and ecology of the tachinids of Central Europe. Mihályi (1986) published a comprehensive identification guide to tachinid genera and species of Hungary. The Siphonini of Europe were revised by Andersen (1996). The Tachinidae chapter of *Manual of Palaearctic Diptera* was authored by Tschorsnig and Richter (1998), the Palaearctic equivalent of Wood’s (1987) chapter in *Manual of Nearctic Diptera*. Chao et al. (1998) reviewed the Tachinidae of China in *Flies of China*, with keys to species and numerous illustrations of external features and male genitalia. The first-ever detailed study of the puparia and larval cephalopharyngeal skeletons of Tachinidae was published by Ziegler (1998). Ziegler, in his phylogenetic conclusions (pp. 192–194), proposed placing Glaurocarini within Ormiini *s. lat.* and placing *Dufouria* Robineau-Desvoidy (type genus of Dufouriini) and *Rondania* Robineau-Desvoidy within Voriini *s. lat.* The decade closed with Sabrosky’s (1999) posthumously published volume on family-group names in Diptera. This work was about 50 years in the making and will be an indispensable reference for decades to come. The Tachinidae with 429 entries dwarfs all other dipteran families.

Traditional taxonomic works of the 21st Century began with a revision of the Polideini of America north of Mexico by O’Hara (2002). There followed a large and well-illustrated work on the identification of Tachinidae of the Russian Far East by Richter (2004). That same year, O’Hara and Wood (2004) published a catalogue of the Tachinidae of America north of Mexico (discussed above). In this work the previous classification of Sabrosky and Arnaud (1965) was revised to conform more closely to the European model of Herting (1984). An interactive online resource to the Tachinidae of Europe was produced by Tschorsnig et al. (2004) as part of the Fauna Europaea project and continues to provide easy access to names and distributions. A catalogue of the Tachinidae of China by O’Hara et al. (2009) provided information on the names, types, distributions, and references of the approximately 1100 species known from this country. The *Manual of Central American Diptera* included a chapter on the Tachinidae by Wood and Zumbado (2010) in which 232 genera were reviewed, keyed, and illustrated (mostly with figures from Wood 1987), thereby forming a fine companion to Wood (1987). A Ph.D. thesis formed the nucleus of Cerretti’s (2010) two-volume work on the Tachinidae of Italy. This treatise provided a wealth of general information on tachinids in addition to generic descriptions and keys to species of Italian Tachinidae. Also included was an interactive key to the tachinid genera of the West Palaearctic Region using the program MOSCH, developed primarily by Cerretti. An online MOSCH key to the tachinid genera of the Palaearctic Region was made available recently by Cerretti et al. (2012a).

The first molecular studies devoted to the Tachinidae made their appearance early in the 21st Century. The Exoristinae were the subject of Stireman’s (2002) molecular study of genes 28S rRNA and EF-1α. The results were only partly congruent with evidence derived from morphology, most notably in not supporting the monophyly of the Goniini. A reappraisal of the same data using a Bayesian analysis (Stireman 2005) did not produce a convincing consensus tree, suggesting that the chosen genes may not be good for inferring tribal relationships within Tachinidae. In a more recent study of the Exoristinae by Tachi and Shima (2010), four genes (white, 18S, 28S and 16S rDNA) were studied. The results were similar in most respects to those of Stireman (2002, 2005), although monophyly of the Goniini was supported. Kutty et al. (2010) examined nine gene regions to infer relationships within the Calyptratae and especially the Oestroidea. In this study their Tachinidae were either monophyletic or not, depending upon the type of analysis performed. In general, these early molecular studies have shown promise and more sophisticated approaches in the future using combined morphological and molecular data sets are expected to yield more convincing results.

## Conclusion

It has been written that to understand the future one must know the past. This is as true of tachinid classification as anything else. The path from Meigen has diverged, joined and meandered to where we are today. Along the way evolutionary thought changed our view of the natural world and the quest to organize animal life then took on new meaning. Chaetotaxy was revealed as an indicator of descent, as were structures of the male and female genitalia. Homoplasy was and continues to bedevil the proper interpretation of tachinid evolution and is the reason why tachinid classification remains unstable. Nevertheless, a great amount of progress has been made in the last 200 years and new technologies are expected to bring about a better understanding of tachinid phylogeny and with it a more stable and predictive classification.

## Footnotes

^1^ Pape et al. (2011) estimated the number of valid species of Tachinidae at slightly over 9600, but this number included *nomina dubia*. Most of the 1300+ *nomina dubia* in the Tachinidae are old names from the 1800s and many of them are likely senior or junior synonyms of other currently recognized species, at least within the well-known fauna of the Palaearctic Region. The number of valid species of Tachinidae without synonymous *nomina dubia* is here roughly estimated at 8500.

^2^ Early authors, beginning with Robineau-Desvoidy (1830), erred in using the name *Tachina* Meigen, 1803 for a genus that should have been called *Exorista* Meigen, 1803 (Sabrosky 1999).

^3^ A long overlooked publication by Meigen (1800) gave *Larvaevora* as an earlier name for *Tachina*. For some years after this discovery family-group names based on *Larvaevora* commonly replaced those based on *Tachina*. The family-group name Larvaevoridae (-inae, -ini) was replaced by Tachinidae (-inae, -ini) when *Larvaevora* Meigen, 1800 was officially suppressed (ICZN 1963).

^4^ When *Salmacia* Meigen, 1800 was suppressed (ICZN 1963), junior synonym *Gonia* Meigen, 1803 took its place. The next available family-group name Goniinae (-ini) replaced that of Salmaciinae (-ini).

^5^ When *Crocuta* Meigen, 1800 was suppressed (ICZN 1963), junior synonym *Siphona* Meigen, 1803 took its place. The next available family-group name Siphonini replaced that of Crocutini.

^6^ Founded on *Echinomya* Latreille, 1804. This name is currently recognized as a junior synonym of *Tachina* Meigen, 1803. The family-group name Tachininae (-ini) has priority over Echinomyiinae (-ini).

^7^ Founded on *Ocyptera* Latreille, 1804. This name is currently recognized as a junior synonym of *Cylindromyia* Meigen, 1803. The family-group name Cylindromyinae (-ini) has priority over Ocypterinae (-ini).

^8^ *Eutachina* Brauer and Bergenstamm, 1889 is currently a junior synonym of *Exorista* Meigen, 1803.

^9^ It was Pantel (1910) who coined the term “microtype” for these tiny ingestible eggs of goniines.

^10^ Mesnil (1956–1965) had called this tribe “Phorocerini oder Exoristini”. Nomenclaturally, Herting’s (1960) use of the name Exoristinae was simply an elevation of Mesnil’s Phorocerini to a subfamily under an alternate name.

^11^ Mesnil (1939) referred to names ending in -inae as tribes and names ending in -ini as subtribes. To avoid confusion within this paper such names are called subfamilies and tribes, respectively.

^12^ Verbeke (1962b) provided a similar discussion of male genitalia as Verbeke (1962a). The latter is more often cited for information that appears in both publications and this convention has been followed here.

^13^ The term Afrotropical was proposed by Crosskey and White (1977) to replace Ethiopian for this biogeographic region. Crosskey (1980a) was chief editor of the voluminous *Catalogue of the Diptera of the Afrotropical Region*.

^14^ Bear in mind that Mesnil (1966) treated all Tachinidae at one rank lower than Crosskey, placing them all in subfamily Tachininae (or Larvaevorinae) with tribes Dexiini, Voriini, Tachinini, Exoristini, Goniini and Phasiini.

^15^ According to Crosskey (1973b: 74), Verbeke (1962a) misidentified *Doleschalla as Torocca Walker*; the true *Torocca* was not studied.
